# A peculiar case of pure intraventricular glioblastoma

**DOI:** 10.1007/s10072-023-06924-4

**Published:** 2023-06-29

**Authors:** Luca Zanuttini, Agnese Orsatti, Matteo Martinoni

**Affiliations:** 1 School of Neurosurgery, Department of Biomedical and Neuromotor Sciences, University of Bologna, Bologna, Italy; 2 IRCCS Institute of Neurological Sciences of Bologna, Division of Neurosurgery, Bellaria Hospital, via Altura 3, 40139 Bologna, Italy; 3 School of Anatomic Pathology, Department of Biomedical and Neuromotor Sciences, University of Bologna, Bologna, Italy; 4 IRCCS Institute of Neurological Sciences of Bologna, Division of Pathology “M. Malpighi”, Bellaria Hospital, Bologna, Italy

**Keywords:** Glioblastoma, Intraventricular glioblastoma, Neurooncology, Neurosurgery, MRI

## Abstract

Glioblastoma (formerly named glioblastoma multiforme) is the most common primary central nervous system tumor, representing 45% of all cases and 15% of all intracranial neoplasms [1]. Its typical radiologic findings and localization make it often a lesion easy to diagnose. In MRI it usually appears as an irregularly shaped cystic lesion with ring contrast enhancement in T1-weighted images, localized in subcortical white matter and deep gray matter nuclei of the cerebral hemispheres. It involves more frequently the frontotemporal region followed by parietal lobes [1]. Few articles in literature described cases of intraventricular glioblastomas, defining those as secondary ventricular tumors because of their probable origin primarily from cerebral tissue with consequent transependymal development [2, 3]. Atypical presentations of these tumors make it more difficult to clearly differentiate them from other lesions more commonly located in the ventricular system. We describe a case with a unique radiological presentation: an intraventricular glioblastoma lying entirely within the ventricular walls, involving all the ventricular system, without mass effect or nodular parenchymal lesions.

A 56-year-old woman with history of ankylosing spondylitis presented with a 2-month drug-resistant headache and short-term memory loss. Brain MRI showed an enhancing multinodular lesion with dissemination along the walls of all the ventricular system (Fig. 1). Total body CT scan excluded other lesions; blood and CSF samples were normal. Following a neurological examination at the admission no focal deficits were found, the patient appeared confused, and presented frequent paraphasias during speech. A frameless stereotactic biopsy was performed. The patient was discharged at postoperative day 4.

**Fig. 1:**
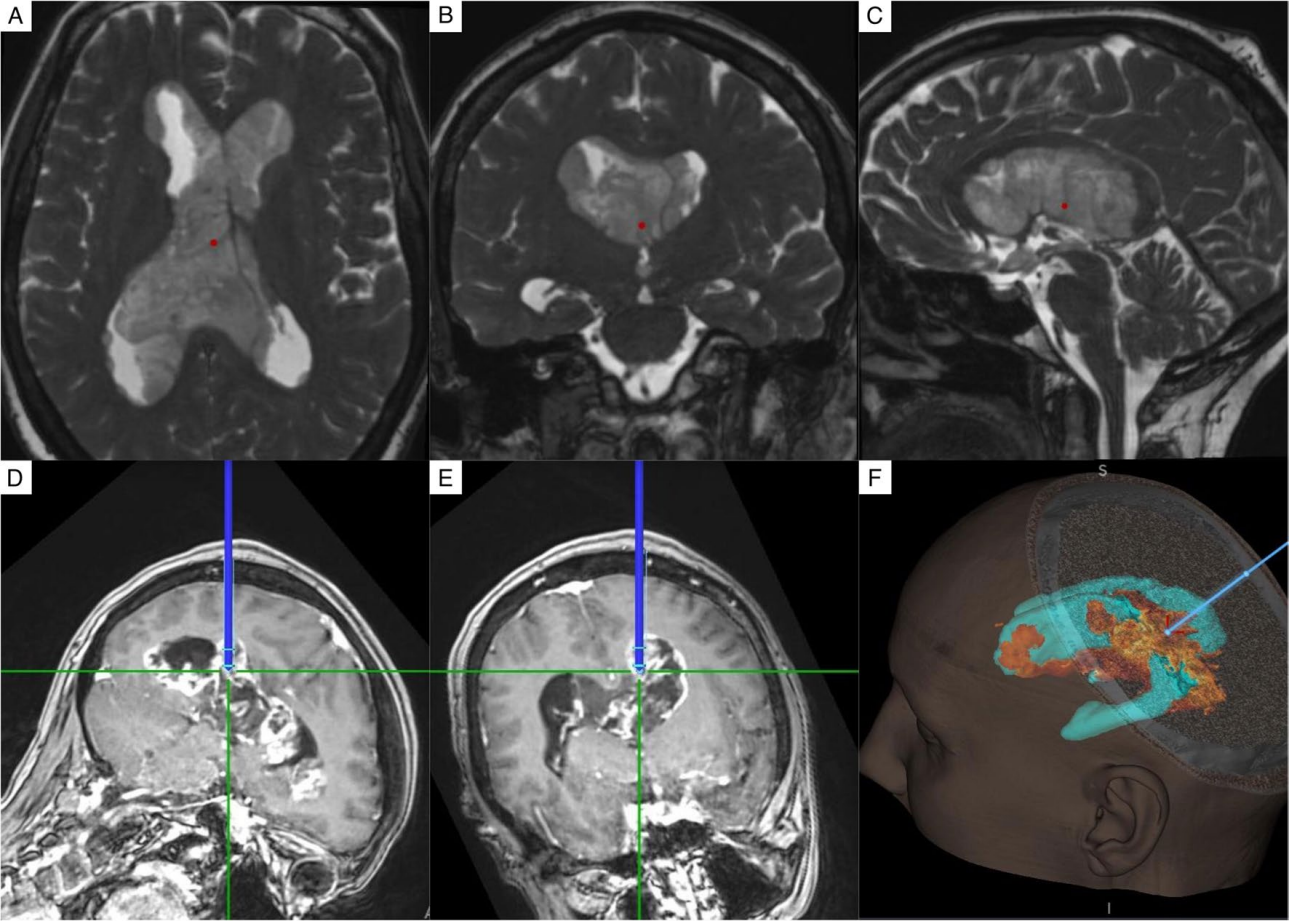
MRI appearance and stereotactic biopsy planning. **A**–**C** T2-heavy weighted high-resolution images showed a purely intraventricular hyperintense lesion compared to white matter, without brain parenchyma involvement. **D, E** Non-homogeneous contrast enhancement appears at T1-weighted images after gadolinium injection. **F** The stereotactic biopsy target was identified in the left ventricular carrefour (the lesion is depicted in orange)

The integrated histological diagnosis with molecular analysis was compatible with glioblastoma IDH-wildtype, grade 4 according to CNS WHO 2021 criteria (Fig. 2) [1].

**Fig. 2:**
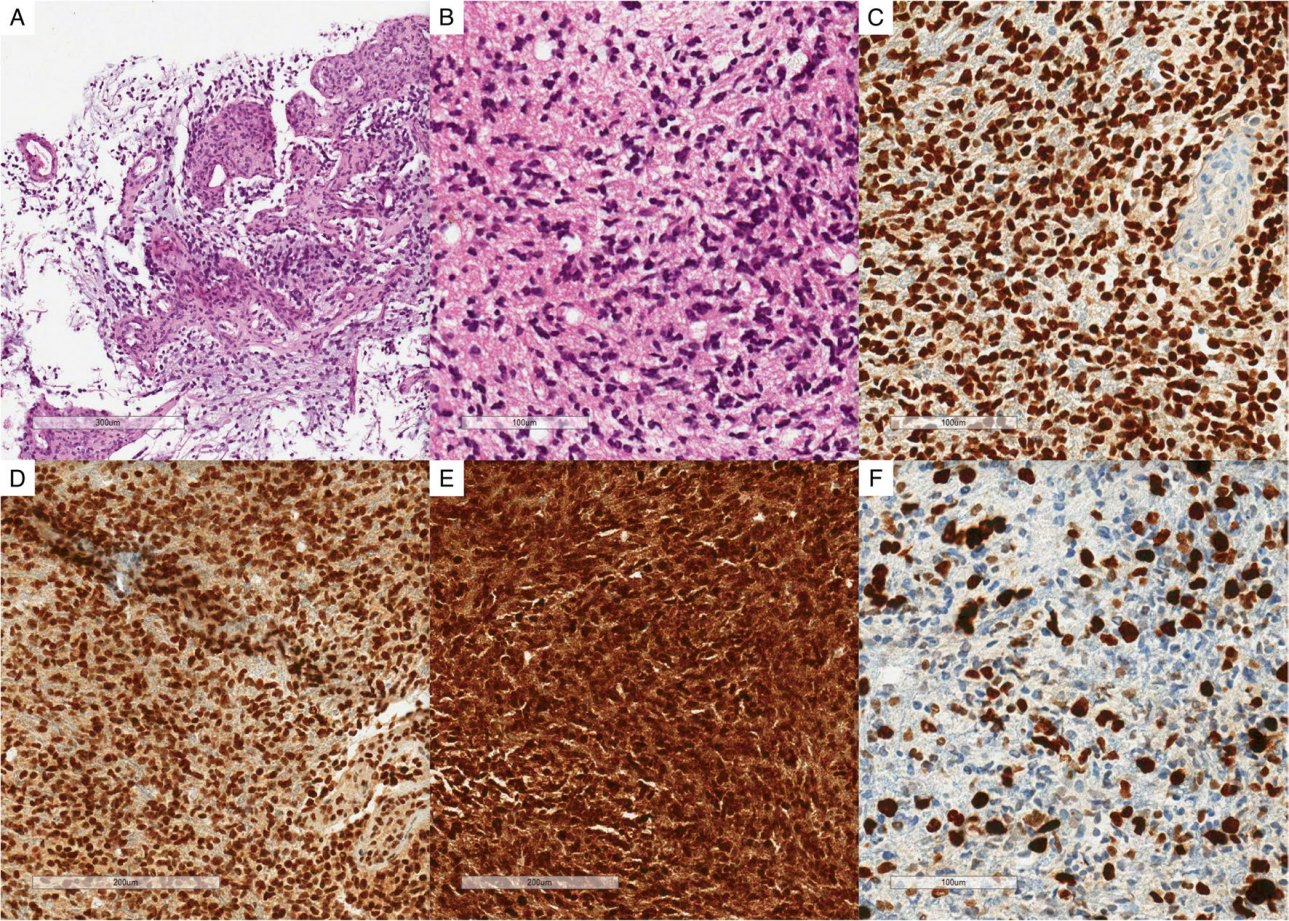
Histological appearance and immunohistochemical staining. **A** Microvascular proliferation, EE (× 10). **B** High-grade tumor area, EE (× 20). **C** OLIG2 positive neoplastic cells on IHC (× 20). **D** ATRX preserved expression on IHC (× 20). **E** P53 hyperexpression detected on IHC (× 20). **F** Ki67 IHC stain showing a high proliferative index in the neoplastic population (18%). Molecular analysis showed absence of IDH and H3 mutation; no TERT promoter mutations were detected; MGMT gene promoter was unmethylated; FISH analysis showed no alterations in EGFR 7p11 and no 7+/10− combination

The case was discussed in the multidisciplinary team: ultimately chemoradiation therapy was not performed because of rapid clinical deterioration of the patient who, in the week following hospital discharge, developed daytime sleepiness, worsening of dysphasia, and loss of autonomy in movements.

Glioblastoma (formerly named glioblastoma multiforme) is the most common primary central nervous system tumor representing 45% of all cases and 15% of all intracranial neoplasms [1]. It usually affects older adults (peak incidence 55–85), having the worst prognosis of all intracranial neoplasms. Most of the affected patients surviving for 15–18 months after chemoradiation [1]. Its most common location is in the subcortical white matter and deep gray matter nuclei, mainly in the frontotemporal region [1].

The peculiar distribution following only the ependymal lining instead of white matter tracts is anecdotic, but it may support the hypothesis that the glioblastomas’ cell of origin (COO) is a neural precursor cell located in the subventricular zone (SVZ) along the lateral ventricular walls. Lee and Manzano stated that possible alternative origins of intraventricular glioblastomas could be the septum pellucidum or the fornix, both components of the limbic system [2]: the involvement of these structures could explain the growing number of amnesic episodes in the clinical history of our case, neurological finding which is, in fact, characteristic of other intraventricular glioblastoma cases reported in literature [3]. However, the most common symptoms associated with intraventricular glioblastomas appear to be related to obstructive hydrocephalus, which surely could be a cause or a concurrent cause to both the initial symptoms of our case.

We here present this case because of its uniqueness: to our knowledge no such cases with subependymal distribution in all ventricular walls, without nodular appearance or mass effect on brain parenchyma, has been described in literature.
